# Sodium Reduction, miRNA Profiling and CVD Risk in Untreated Hypertensives: a Randomized, Double-Blind, Placebo-Controlled Trial

**DOI:** 10.1038/s41598-018-31139-5

**Published:** 2018-08-24

**Authors:** Li Chen, Feng J. He, Yanbin Dong, Ying Huang, Gregory A. Harshfield, Haidong Zhu

**Affiliations:** 1 Georgia Prevention Institute, Department of Population Health Sciences, Medical College of Georgia, Augusta University, Augusta, Georgia USA; 2 Wolfson Institute of Preventive Medicine, Barts and The London School of Medicine & Dentistry, Queen Mary University of London, London, UK

## Abstract

Sodium reduction decreases blood pressure (BP) and cardiovascular mortality. However, the underlying molecular mechanisms are not well understood. We tested the hypothesis that reduction of sodium intake would change miRNA expression in hypertensive patients, and those changes would be associated with improved cardiovascular phenotypes. A whole genome RNA sequencing was performed in paired serum samples collected at the end of usual sodium intake and reduced sodium intake periods from 10 (age 56.8 ± 8.9) untreated black male hypertensives, selected from a randomized crossover trial of sodium reduction as the discovery cohort. Validation was carried out by the PCR Serum/Plasma Focus panel profiling in paired samples in all 64 (50% males, age 50.2 ± 9.5) untreated black hypertensives from the same trial. Fifteen respondent miRNAs were identified in the discovery stage. miR-143-3p was replicated. Sodium reduction up-regulated miR-143-3p. The increase in miR-143-3p was associated with the reduction of BP and arterial stiffness and the increase in skin capillary density. In conclusion, dietary sodium reduction alters circulating miRNA expressions, and those miRNA changes are associated with reduced BP and improved arterial compliance in untreated black hypertensives, suggesting that miRNA regulation may be one of the underlying mechanisms that dietary sodium regulates cardiovascular health.

## Introduction

High sodium intake is a significant risk factor for cardiovascular disease (CVD). Dietary sodium reduction has been shown to reduce blood pressure (BP), the incidence of CVD and mortality^1–4^. In spite of these well-established relations, the underlying biological mechanisms are not well understood.

miRNAs, small non-coding RNA molecules of 16–22 nucleotides, are post-translational regulators by targeting mRNA and regulating the mRNA stability and their translation, which, in turns, regulate cellular function and biological process. Aberrant regulation of miRNA expression has been shown to be involved in pathological events underlying hypertension and CVD both in animal experiments and human studies^5–11^. Moreover, animal studies demonstrate that miRNAs play a role in high sodium intake-induced myocardial fibrosis and cardiac hypertrophy, Ang II signaling and salt-sensitive hypertension^5–7,12^. However, studies examining the effects of dietary sodium intake on miRNA regulation in humans are limited.

We previously conducted a randomized double-blind, placebo-controlled crossover trial of dietary sodium reduction in untreated hypertensives, showing that reduction in sodium intake reduced BP, urinary albumin excretion and improved arterial compliance^13^. In this study, we tested the hypothesis that reduction in dietary sodium intake would induce changes in miRNA expression among black hypertensive subjects, given that black populations have higher prevalence of hypertension and CVD, and are more likely to be salt sensitive. We further examined whether changes in miRNA expression would be associated with reduced BP, urinary albumin excretion, and improved arterial compliance.

## Results

### Participants Characteristics

The 10 black males, selected into the discovery stage, had a mean age of 56.80 ± 8.91 years and mean BMI 39.79 ± 6.71 kg/m^2^ (Table 1). All black subjects with serum samples available were included in the validation stage (N = 64), with mean age of 50.17 ± 9.52 years, mean BMI 30.88 ± 5.31 kg/m^2^ and 50% were males (Table 1). Both in the discovery cohort and in the validation cohort, sodium reduction lowered BP, urinary sodium excretion, increased plasma renin activity (PRA) and increased capillary density (*p*s < 0.05), as we previously reported^13^.

**Table 1:** General characteristics of participants.

Characteristic	Discover (N = 10)			Validation (N = 64)		
	Sodium	Placebo	*p*	Sodium	Placebo	*p*
Age (years)^†^	56.80 ± 8.91	—	—	50.17 ± 9.52	—	—
Male (%)	10 (100)	—	—	32 (50)	—	—
BMI (kg/m^2^)^†^	39.79 ± 6.71	—	—	30.88 ± 5.31	—	—
Office BP and pulse rate						
SBP (mmHg)	155.10 ± 14.09	141.50 ± 12.14	<0.001	148.89 ± 13.47	143.66 ± 11.82	<0.001
DBP (mmHg)	91.50 ± 10.83	86.10 ± 10.51	0.002	90.72 ± 9.14	88.23 ± 9.11	<0.001
MAP (mmHg)	112.70 ± 10.71	104.57 ± 9.88	<0.001	110.11 ± 9.38	106.71 ± 8.95	<0.001
Pulse rate (beat/min)	63.40 ± 11.74	67.30 ± 13.61	0.162	67.63 ± 9.92	67.23 ± 9.90	0.690
Ambulatory BP						
24-hour SBP (mmHg)	145.60 ± 9.47	136.90 ± 9.04	<0.001	144.54 ± 8.43	140.08 ± 10.09	<0.001
24-hour DBP (mmHg)	90.20 ± 10.51	85.70 ± 9.64	0.004	87.75 ± 8.86	85.37 ± 8.99	<0.001
Day SBP	151.60 ± 9.03	141.80 ± 8.73	<0.001	150.07 ± 9.25	144.90 ± 10.37	<0.001
Day DBP	94.70 ± 11.22	89.30 ± 9.82	0.005	92.74 ± 9.68	89.90 ± 9.64	<0.001
Night SBP	138.10 ± 12.59	131.4 ± 11.35	0.005	138.20 ± 9.69	134.14 ± 12.08	<0.001
Night DBP	84.50 ± 10.87	81.70 ± 11.52	0.089	82.36 ± 9.27	80.37 ± 9.83	0.017
Urinary measurements						
Volume (mL/24 hours)	2027.60 ± 462.94	1588.00 ± 374.52	0.012	1568.81 ± 575.20	1553.85 ± 630.89	0.818
Sodium (mmol/24 hours)	218.01 ± 58.43	110.45 ± 31.97	<0.001	163.14 ± 60.72	115.24 ± 43.64	<0.001
Potassium (mmol/24 hours)^*^	79.97 ± 17.72	72.05 ± 18.46	0.075	64.24 ± 27.59	62.23 ± 18.79	0.738
Creatinine (mmol/24 hours)^*^	17.89 ± 3.70	17.54 ± 4.00	0.647	14.41 ± 4.80	15.02 ± 4.65	0.177
Albumin (ug/24 hours)^*^	66167.34 ± 145070.90	30160.16 ± 39208.77	0.093	26839.18 ± 70816.02	15200.83 ± 18366.33	0.048
Albumin/creatinine ratio (ug/mmol)^*^	3888.90 ± 7982.49	1677.19 ± 1795.45	0.139	2090.42 ± 4756.87	1179.28 ± 1211.20	0.001
Pulse wave velocity (m/s)	11.92 ± 1.40	11.53 ± 1.54	0.326	11.75 ± 2.07	11.21 ± 1.77	0.003
Plasma measurements						
Renin activity (ng/mL/h)^*^	0.20 ± 0.16	0.37 ± 0.35	0.017	0.19 ± 0.24	0.23 ± 0.33	0.016
Aldosterone (pmol/L)	271.50 ± 94.28	329.90 ± 140.46	0.116	301.41 ± 135.85	333.58 ± 158.06	0.105
Capillary density by capillaroscopy (capillaries/mm^2^)						
Dorsum of the fingers						
At resting	64.39 ± 16.99	67.07 ± 16.54	0.532	67.59 ± 14.99	70.49 ± 16.02	0.053
With venous congestion	68.43 ± 13.34	74.86 ± 15.18	0.169	70.42 ± 14.76	76.58 ± 16.23	<0.001
Side of the fingers						
At resting	58.19 ± 20.62	65.31 ± 19.54	0.058	63.90 ± 16.23	68.34 ± 17.76	<0.001
With venous congestion	60.50 ± 20.38	68.50 ± 18.20	0.104	69.02 ± 17.44	73.39 ± 18.81	0.005
Capillary density by OPS imaging (capillaries/mm^2^)						
Dorsum of the fingers	26.09 ± 4.82	28.94 ± 5.81	0.002	29.67 ± 6.72	30.56 ± 7.04	0.251
Web of the hand	26.22 ± 8.88	26.89 ± 4.86	0.869	22.36 ± 6.31	25.18 ± 5.09	0.016
Estimated glomerular filtration rate (mL/min/1.73 m^2^)	90.81 ± 15.93	86.74 ± 16.74	0.223	94.89 ± 17.45	93.36 ± 19.29	0.228

^*^Wilcoxon matched-pairs signed-ranks test was used.

^†^Age and BMI only measured once.

### Differential miRNA expression discovered by next-generation RNA-sequencing

On average, 10.2 million reads were obtained from each sample. After mapping the data and counting to relevant entries in miRBase 20, the number of known miRNAs was calculated. A total of 327 miRNAs were identified in all samples with average transcripts per kilobase million (TPM) ≥1, and 214 miRNAs were identified with TPM ≥5. There were 15 miRNAs differently expressed (raw *p*s < 0.05) with TPM ≥5 (Table 2).

**Table 2:** Effects of sodium reduction on miRNAs expression.

Rank	miRNA	Discover (N = 10)			Validation (N = 64)				
		Beta^‡^	*p*	FDR	Sodium^*^	Placebo^*^	Delta^†^	Beta^‡^	*p*
1	miR-425-3p	−0.44	0.002	0.175	−5.80	−5.66	0.14	−0.01	0.919
2	miR-576-3p	0.58	0.004	0.226					
3	miR-148a-5p	0.82	0.006	0.299					
4	miR-143-3p	0.63	0.007	0.341	−4.53	−4.47	0.06	0.32	0.030
5	miR-424-3p	0.36	0.012	0.397					
6	miR-30e-5p	0.31	0.014	0.430	−1.98	−2.04	−0.06	0.01	0.912
7	miR-27a-5p	0.35	0.022	0.503					
8	miR-148a-3p	0.42	0.025	0.524	−3.43	−3.76	−0.33	0.06	0.704
9	miR-142-5p	−0.68	0.029	0.524	−5.44	−5.47	−0.04	−0.31	0.060
10	miR-193b-5p	0.38	0.029	0.524					
11	miR-4433b-3p	0.25	0.031	0.524					
12	miR-4433b-5p	−0.76	0.035	0.524					
13	miR-193a-5p	0.36	0.035	0.524	−7.84	−7.74	0.09	0.25	0.159
14	miR-223-5p	0.40	0.039	0.524	−7.15	−6.91	0.25	−0.49	0.023
15	miR-19b-3p	0.42	0.046	0.569	1.25	1.12	−0.12	−0.05	0.643

^*^The unit of miRNA expression was normalized quantification cycle (dCq, average Cq – assay Cq) in the validation profiling.

^†^Delta: Unadjusted changes of miRNA expression from sodium tablets to placebo tablets.

^‡^Beta: Regression coefficient of measurement type in the mixed-effects model adjusted for age, sex and BMI, reflects miRNA expression change from sodium tablets to placebo tablets.

### Validation by PCR Serum/Plasma Focus panel

Validation of RNA-seq findings was done by using a different platform (i.e. RT-PCR) and in larger sample size. To maximize the chance for validation and minimize false positive results, we selected 8 miRNAs that are highly expressed in the circulation for validation by using PCR Serum/Plasma Focus panel (total 179 miRNAs). Validation was defined as raw *p* < 0.05 and consistent directionality with discovery. Although the *p*-value of miR-223-5p is 0.023, the direction was opposite to discovery. Only miR-143-3p (*p* = 0.030) was successfully validated (β = 0.32, *p* = 0.030), that is sodium reduction increased miR-143-3p expression (Table 2).

### Association of changes in miRNAs with changes in CVD phenotypes

Next, we examined the relationship between change in miR-143-3p and changes in CVD phenotypes in our study. Up-regulated miR-143-3p was associated with reduced ambulatory daytime SBP (β = −1.92 *p* = 0.023). Moreover, up-regulated miR-143-3p was associated with reduced carotid-femoral pulse wave velocity (PWV) (β = −0.30, *p* = 0.031, Table 3) and with increased skin capillary density (β = 2.89, *p* = 0.026, Table 3).

**Table 3:** Association between changes in miRNAs and changes in CVD measurements.

Phenotype	miR-143-3p	
	β	*p*-value
24 h urinary sodium excretion	0.32	0.030
Office BP		
SBP	−0.05	0.959
DBP	−0.64	0.267
MAP	−0.50	0.460
Ambulatory BP		
24-hour SBP	−1.12	0.156
24-hour DBP	−0.74	0.186
Day SBP	−1.92	0.023
Day DBP	−1.00	0.116
Night SBP	−0.13	0.891
Night DBP	−0.39	0.555
Urinary measurements		
Albumin^*^	−1.74E-04	0.645
Albumin/creatinine ratio^*^	−4.79E-04	0.773
Pulse wave velocity	−0.30	0.031
Plasma measurements		
Renin activity^†^	0.03	0.621
Aldosterone	−0.25	0.985
Skin temperature	−0.03	0.890
Capillary density by capillaroscopy		
Dorsum of the fingers		
At resting	1.68	0.156
With venous congestion	2.89	0.026
Side of the fingers		
At resting	0.87	0.433
With venous congestion	0.81	0.523
Capillary density by OPS imaging		
Dorsum of the fingers	0.03	0.956
Web of the hand	0.03	0.974
Estimated glomerular filtration rate	−0.56	0.594

^*^Inverse-root transformed; ^†^Log transformed.

## Discussion

The present study showed that dietary sodium reduction increased circulating miR-143-3p level in untreated black hypertensives. Moreover, the increase in miR-143-3p was associated with the reduction of BP and arterial stiffness and the increase in skin capillary density.

A few studies have profiled miRNA expression in salt sensitive individuals, however, the results are inconsistent, possibly due to different populations studied^14,15^. To the best of our knowledge, our study is the first to examine the effect of reduction in dietary sodium intake on miRNA expression in untreated hypertensive patients. In the present study, miR-143-3p was up-regulated in the circulation by sodium reduction, and the increase in miR-143-3p expression was associated with the reduced daytime ambulatory SBP and the improvement of arterial stiffness and skin capillary density, which may slow down vascular aging.

miR-143 is expressed in various tissues including vascular smooth muscle cells (VSMC), heart, aorta, lung, skeletal muscle, fat and skin^16^. miR-143 is highly expressed in the heart and aorta and is considered a cardiovascular-specific miRNA. Circulating miRNA levels could result from passive release or active secretion and serve as signaling molecules influence systemic process^17^. Circulating levels of miR-143-3p were lower in obese individuals compared with lean subjects^18^. A significant decrease in the expression of miR-143 was found in aorta of patients having an aortic aneurism^19^. Our findings echo the earlier observation that miR-143-3p was associated with lower 24-hour ABP^8^.

We further showed that increased circulating miRNA-143-3p level by sodium reduction was also associated with reduced carotid-femoral PWV and increased skin capillary density. Carotid-femoral PWV is a surrogate marker of arterial stiffness, which is a strong predictor of future cardiovascular events and all-cause mortality^20^. Microvascular rarefaction, a reduction in the number of arterioles and capillaries, has been found in many animal models of hypertension and human hypertension. Microvascular rarefaction increases peripheral vascular resistance, leading to increased BP and target organ damage. Reduced miR-143-3p expression by sodium reduction was associated with increased capillary density in the present study.

The mechanisms how miR-143-3p are involved in BP regulation, arterial stiffness and microvascular rarefaction are not fully understood. miR-143-3p has been shown to regulate ACE2 gene expression, which is involved in BP regulation^10^. miR-143 and miR-145 acted as molecular keys to switch the phenotypes of VSMCs, regulating VSMC proliferation and differentiation, actin modeling, contractility and podosome formation and migration^16^. miR-143-3p can suppress extracellular matrix (ECM) proliferation and ECM protein deposition in TGF-β1-mediated airway smooth muscle cells^21^. Recent evidence showed that higher miR-143-3p stimulates glucose update and increases insulin sensitivity^22^, which may be an underlying mechanism for reduced BP and improved arterial stiffness seen in our study. An intervention study in animal model is needed to establish this mechanistic link.

The strengths of our study include that we utilized the sample from a well-controlled, randomized, double-blind clinical trial of dietary sodium reduction with well-characterized CVD phenotypes. In addition, we focused on black hypertensives, in whom salt sensitivity and salt-sensitive hypertension are common. However, our effect size was small, which could be due to the modest sample size and our finding could be a chance finding. Future studies are warranted to replicate our findings in large independent cohorts and in different populations. We acknowledge that 8 out of 15 miRNAs were selected for validation because of their higher expression in the circulation. The remaining 7 miRNAs need to be validated in larger studies. Very few studies studied the effects of dietary sodium on miRNA regulation. Our study contributes to the ongoing effort to determine the impact of dietary sodium on epigenetic regulation and its role in BP regulation and cardiovascular health.

## Methods

### Participants

The present study utilized stored serum samples from a previously conducted randomized double-blind, placebo-controlled crossover trial of dietary sodium reduction in untreated hypertensives. The inclusion criteria was population aged 30 to 75, with sitting systolic BP 140 to 170 mmHg or diastolic 90 to 105 mmHg, and with no previous treatment for raised BP^13^. The present study was a two-stage design. The discovery cohort consisted of 10 selected black males. In order to maximize the chance for discovery, the selection was based on the most significant changes on urinary sodium excretion and black males only. The validation cohort included all black participants with serum samples available at both time points (end of slow sodium and placebo). The study was approved by the Wandsworth Local Research Ethnics Committee and the Institutional Review Board of Augusta University. Informed consent was obtained from all participants. Our study was performed in compliance with the principles of the Declaration of Helsinki of the World Medical Association.

### Sodium reduction protocol

A randomized, placebo-controlled, double-blind crossover trial was carried out. In the first two weeks, all participants were advised to adopt reduced-sodium intake aiming at achieving sodium daily intake around 2.0 grams. Detailed advice was given by trained nurses at the beginning and reinforced at each visit to the participants, and when appropriate to the spouse or whoever cooked in the household. While continuing on the reduced sodium diet, participants were given, in a random order, either slow sodium tablets (10 mmol sodium per tablet) or nine placebos daily for six weeks. They then crossed over to receive the other tablets for another six weeks^13^. Serum samples together with other anthropometric and laboratory measurements were taken at the end of each six-week period. Reduced-sodium diet plus slow sodium tablets represented normal sodium intake, while reduced-sodium diet plus placebo represented a reduced-sodium diet. miRNA levels and other measurements taken after six weeks sodium tablet period were compared with the ones 6 weeks after placebo period to study the effect of sodium reduction.

### Anthropometric and laboratory measurements

Height and body weight were measured with light clothing and without shoes. Body mass index (BMI) was calculated as weight (kg) per square of height (m^2^). BP was measured by a validated automatic digital BP monitor (Omron HEM-705CP) in sitting position after 5 to 10 minutes rest. Three readings were taken and the average of the last two readings was used. Twenty-four-hour ambulatory blood pressure monitoring (ABPM) was performed using a SpaceLabs 90207 device (SpaceLabs, InC, Washington, DC) as previously described^23^. Briefly, monitoring was set to take measurements at half hourly intervals during the day and hourly intervals overnight. Recordings were analyzed with the ambulatory BP monitoring report manager system software package^23^. Blood samples were taken for measurements of routine biochemistry. Two consecutive 24-hour urines were collected for measurements of urinary albumin. Carotid-femoral pulse wave velocity (PWV) was measured noninvasively using an automatic device Complior^13^. Estimated glomerular filtration rate (eGFR) was estimated by CKD-EPI creatinine equation^24^.

$${\rm{eGFR}}=141\times \,{\rm{\min }}\,{(\frac{{{S}}_{{Cr}}}{{\kappa }},1)}^{\alpha }\times \,{\rm{\max }}\,{(\frac{{{S}}_{{Cr}}}{{\kappa }},1)}^{-1.209}\times {0.993}^{{Age}}\times \beta \times \gamma $$

where *S*_*cr*_ is the standardized serum creatinine, $\kappa$ equals to 0.7 in females or 0.9 in males, $\alpha$ equals to −0.329 in females or −0.411 in males, $\beta$ equals to 1.018 in females or 1 in males, and *γ* equals to 1.159 in Black or 1 in White.

### Skin Capillary Density Measurement

Intravital Capillaroscopy Skin capillary density was measured according to a standardized technique as described previously^25^. Microscopic images were obtained with a charge coupled device camera (Sony model XC-75CE) and were stored using a video recorder (JVC model HR-S6600). The skin of the dorsum and the side of the middle phalanx of the left hand were examined. Four microscopic fields (0.66 mm2 per field) centered on an ink spot at each site were recorded continuously for 5 minutes to detect intermittently perfused capillaries. The number of capillaries per field was counted online and by running the recorded tapes using computer software (CapiScope, KK-Technology). To maximize the number of visible capillaries, venous congestion was carried out. A miniature neonatal BP cuff was applied to the base of the left middle finger. The cuff was inflated and maintained at 60 mm Hg for 2 minutes. During the venous congestion, further images were recorded using 1 of the 4 microscopic fields chosen at random. We used Cytoscan video microscopy to examine skin capillary density. Images from 4 random fields of the dorsum of the middle phalanx of the left hand were stored. In addition, 3 fields from the webbed skin between the index and middle finger, and the ring and middle finger, as well as the little and ring finger, were obtained. Each field (0.26 mm2) was continuously recorded for 3 minutes to identify intermittently perfused capillaries. Images were stored on a video recorder (JVC model HR-S6600). The number of capillaries per field was counted offline using computer software (KK-Technology).

### Extract RNA from serum samples

Total RNA was extracted from 500 µL serum samples using miRCURY RNA Isolation Kits-Biofluids (Exiqon, Vedbaek, Denmark). Exiqon’s RNA spike-in kit for quality control of the RNA isolation was applied. Three RNA isolation controls (UniSp2, UniSp4 and UniSp5) pre-mixed, each at different concentration in 100 fold increments were added to the purification to detect any differences in extraction efficiency. We observed an excellent correlation of counts corresponding to the spike-ins between the samples.

### Library preparation and Next-Generation Sequencing (NGS)

Samples were sequenced on the Illumina NextSeq. 500 system. A total of 6 ul of total RNA was converted into microRNA NGS libraries using NEBNEXT library generation kit (New England Biolabs Inc.). Each individual RNA sample had adaptors ligated to its 3′ and 5′ ends and converted into cDNA using miRCURY LNA Universal RT microRNA PCR kit (Exiqon). Then the cDNA was pre-amplified with specific primers containing samples specific indexes. After 18 PCR cycles, the libraries were purified on QiaQuick columns and the insert efficiency evaluated by Bioanalyzer 2100 instrument on high sensitivity DNA chip (Aglient Inc.) The miRNA cDNA libraries were size fractionated on a LabChip XT (Caliper Inc.) and a band representing adaptors and 15-40 bp insert excised. Samples were then qualified using qPCR and concentration standards. Based on the quality of the inserts and the concentration measurements the libraries were pooled in equimolar concentrations. The library pool (s) were finally quantified again with qPCR and optimal concentration of the library pool used to generate the clusters on the surface of a flow cell before sequencing using v2 sequencing methodology (Illumina InC.). All experiments were conducted at Exiqon Service, Denmark. Expression levels were measured as Tags Per Million (TPM), which is the number of reads for a particular miRNA divided by the total number of mapped reads and multiplied by 1 million.

### Validation by miRNA profiling

We validated the RNA-seq findings by applying quantitative real-time polymerase chain reaction (RT-PCR) method in all black participants with available serum samples (n = 64) at both time points (end of slow sodium and end of placebo) using Exiqon service. Each RNA sample was poly-adenylated and successfully reversely transcribed into cDNA in a single reaction step. cDNA and ExiLENT SYBR Green master mix were transferred to the miRCURY LNA TM Universal RT microRNA PCR Serum/Plasma Focus panel pre-loaded with primers, using a pipetting robot. Amplification was performed in a Roche Lightcycler 480. The cDNA synthesis control one spike-in (UniSp6) was added in the reverse transcription reaction to confirm that the reverse transcription and amplification occurred with equal efficiency in all samples. In addition, a DNA spike-in (UniSp3) was present on all panels. The DNA spike-in consists of a premixed combination of DNA template and primers. Deviation in this reaction will indicate inhibition at the qPCR level. The amplification curves were analyzed using the Roche LC software, both for determination of quantification cycle Cq (by the 2nd max derivative method) and for melting curve analysis. The amplification efficiency was calculated using algorithms similar to the LinReg software. All assays were inspected for distinct melting curves and the Tm was checked to be within known specifications for the assay. Furthermore, assays had to be detected with 5 Cqs less than the negative control, and with Cq < 37 to be included in the data analysis. Data that did not pass these criteria were omitted from any further analysis. Cq was calculated as the 2^nd^ derivative. All samples passed the quality control analysis and no signs of PCR reaction inhibition or hemolysis were observed. The expression levels were comparable for all samples included in the analysis, and within the detection limit of the system. All 179 miRNAs on the Serum/Plasma Focus panel (Qiagen/Exiqon, cat# YAHS-106Y) were detected. On average, 146 miRNAs were detected per sample. For normalization of the data, the average of the assays detected in all samples (n = 128) was applied as this is found to be the most stable normalizer. Normalized Cq = average Cq (n = 128) – assay Cq (sample). The normalized Cq (dCq) values were used in the analysis.

### Statistical analysis

The general characteristics of the subjects are presented as mean ± standard deviation (SD) for continuous variables and N (%) for categorical variables. Normality of each continuous variables were tested based on a combination test statistics of skewness and kurtosis. Two-tailed paired t-test was conducted to examine the differences of variables with normal distribution between placebo and sodium tablets. Wilcoxon matched-pairs signed-ranks test was used to test for non-normal distributed variables. Mixed-effects linear regression was used to assess the differential expression of miRNAs between sodium and placebo tablets while incorporating repeated measured data and controlling for age, sex and BMI as confounding variables. To correct for multiple testing, the set of raw *p*-values was converted to FDRs according to Bonferroni’s correction^26^. A *p*-value < 0.05 was considered statistically significant.

We further tested whether the change in the validated miR-143-3p was associated with changes in CVD outcomes. Mixed-effects regression was also used to examine the association between miRNA expression levels and cardiovascular phenotypes. Variables included systolic blood pressure (SBP), diastolic blood pressure (DBP), 24-hour SBP and DBP, daytime SBP and DBP, night SBP and DBP, urine albumin (UAlb, inverse-root transformed), urine albumin/creatinine ratio (UAlb/Cr, inverse-root transformed), PWV, PRA (log transformed), aldosterone and capillary density measurements. Tests for normality based on skewness and kurtosis were used for selected variables^27^, and transformations were made to convert the variables into normally distributed variables if necessary. All analyses were performed using Stata version 12.0 (StataCorp., College Station, Texas, USA) and R version 3.3.3 (R Foundation for Statistical Computing Vienna, Austria).

## Data Availability

The datasets generated during the current study are available from the corresponding author on request.
